# Association of *SPP1* and *NCAPG* genes with milk production traits in Chinese Holstein cows: polymorphism and functional validation analysis

**DOI:** 10.3389/fvets.2024.1435128

**Published:** 2024-10-30

**Authors:** Chuanchuan Wang, Yafei Chen, Jinyan Zhao, Xiaofang Feng, Ruoshuang Ma, Hua Wang, Lin Xue, Jinli Tian, Lijuan Yang, Yaling Gu, Juan Zhang

**Affiliations:** ^1^College of Animal Science and Technology, Ningxia University, Yinchuan, China; ^2^Key Laboratory of Ruminant Molecular Cell Breeding in Ningxia, Ningxia University, Yinchuan, China; ^3^Yinchuan Animal Husbandry Technical Extension and Service Center, Yinchuan, China

**Keywords:** *SPP1*, *NCAPG*, milk production traits, association analysis, SNP, functional verification

## Abstract

Milk production traits play an important role in dairy cattle breeding, and single nucleotide polymorphisms can be used as effective molecular markers for milk production trait marker-assisted breeding in dairy cattle. Based on the results of the preliminary GWAS, candidate genes *SPP1* and *NCAPG* associated with milk production traits were screened. In this study, the aim was to screen and characterize the SNPs of *SPP1* and *NCAPG* genes about milk production traits. Two SNPs and one haplotype block of the *SPP1* gene and four SNPs and one haplotype block of the *NCAPG* gene were obtained by amplification, sequencing and association analysis, and all six SNPs were located in the exon region. Association analysis showed that all six SNPs were significantly associated with milk protein percentage. Linkage disequilibrium analysis showed that 2 SNPs of *SPP1* (g. 36,700,265 C > T and g. 36,693,596 C > A) constituted a haplotype that correlated with milk protein percentage, and the dominant haplotype was H2H2, which was CCTT. 4 SNPs of *NCAPG* (g. 37,342,705 C > A, g. 37,343,379 G > T, g. 37,374,314 C > A and g. 37,377,857 G > A) constituted a haplotype associated with milk protein percentage, 305-days milk protein yield and 305 days milk yield. Tissue expression profiling results revealed that *SPP1* and *NCAPG* had the highest expression in mammary tissue. Interference with *SPP1* and *NCAPG* inhibited the proliferation of Bovine mammary epithelial cells. (BMECs), down-regulated the expression of *PCNA*, *CDK2* and *CCND1*, up-regulated the expression of *BAX* and *BAD*, and promoted apoptosis. Reduced triglyceride synthesis in BMECs, down-regulated the expression of *DGAT1*, *DGAT2*, *LPIN1*, and *AGPAT6*.*SPP1* and *NCAPG* are involved in the synthesis of milk proteins, and interfering with *SPP1* and *NCAPG* decreased the secretion of *β*-casein, *κ*-casein, and αs1-casein, as well as up-regulated the *CSN2* and *CSN3* expression. The above results indicate that the SNP loci of *SPP1* and *NCAPG* can be used as potential molecular markers to improve milk production traits in dairy cows, laying the foundation for marker-assisted selection. It also proves that *SPP1* and *NCAPG* can be used as candidate key genes for milk production traits in dairy cows, providing new insights into the physiological mechanisms of lactation regulation in dairy cows.

## Introduction

Milk is considered a food of high nutritional value, rich in proteins, fats, carbohydrates, minerals, and vitamins, as well as free fatty acids and conjugated linoleic acid, which meet the nutritional needs of the developing individual. High milk yield is one of the most important traits in global dairy breeding, and milk composition characterization is a key breeding goal to meet the needs of a healthier human diet, with protein considered one of the most valuable components (1). Milk proteins are categorized according to their solubility into casein (about 80%), whey proteins (about 14%) and fat globule membrane proteins (about 6%) (2). In the last few years, numerous studies have been conducted on polymorphisms in milk production candidate genes, especially the relationship between allelic variation and certain milk production traits (milk yield, milk fat percentage, milk protein percentage) (3). In traditional breeding work, the selection and breeding of milk production traits in dairy cows is slow. Therefore, molecular marker-assisted breeding is a new situation in the current breeding work. Single nucleotide polymorphisms (SNPs) in DNA molecular genetic markers have been widely used, and the prospect is very promising (4).

Dairy Herd Improvement (DHI), a technique for measuring milk production performance and milk composition in dairy cows. In our previous study, DHI data of Holstein dairy cows in Ningxia region were collected from 2012 to 2019, involving a total of about 50,000 cows from 30 large-scale dairy farms, using corrected phenotypic data for milk production traits and somatic cell scores (SCS), and individuals with phenotypes were genotyped using the Illumina BovineSNP150 BeadChip, and genome-wide linkage analyses were conducted by using FarmCPU for the screening of important SNPs and candidate genes (5). With the rapid advancement of gene chips, high-throughput sequencing and histological big data technologies, the cost of typing detection of SNP markers has been dramatically reduced, which has led to a wave of development of livestock and poultry breeding from the traditional BLUP method to genome-wide selection breeding. Based on the results of GWAS, we used GO and KEGG for enrichment analysis and selected Secreted phosphoprotein 1 (*SPP1*) and Non-SMC Condensin I Complex Subunit G (*NCAPG*) for association analysis of SNPs for milk production traits in Holstein cows. Increasingly, SNPs are important for milk production, growth, reproduction and other traits in cattle (6).

*SPP1*, also known as osteobridging protein (OPN), is located on chromosome 6 NC_037333.1 (36693204.36700208, complement), was initially found in osteoblasts and has been implicated in a variety of pathogenetic mechanisms including insulin resistance, inflammation and bone remodeling (7, 8). *SPP1* is highly expressed in the kidney, liver, mammary tissue, immune cells and salivary glands (9). It is a multifunctional phosphorylated protein involved in a variety of biological activities such as stimulation of brain, gut and immune development (10). *SPP1* binds to receptor integrins with multiple functions, thereby activating cell signaling pathways, such as PI3K/Akt and MAPK signaling cascades, to exert its multiple functions (11, 12). The main sources of *SPP1* are mammary epithelial cells and monocytes and macrophages in milk. Bone-bridging proteins were detected in raw milk from cows at a concentration of 8 mg/L (13). The presence of *SPP1* in milk and its high expression in mammary tissues may be responsible for the proliferation and differentiation of recipient BMECs. Schnabel et al. showed (14) that *SPP1* was associated with milk protein rate in dairy cows. Four SNPs were identified in a region approximately 5 kb upstream of bovine *SPP1*, These SNPs are specifically *SPP1*c.-1301G > A, *SPP1*c.-1251C > T, *SPP1*c.-430G > A, and *SPP1*c.*40A > C, where *SPP1*c.-1301G > A is associated with milk fat percentage and milk protein percentage. In Czech Fleckvieh cattle, SNPs in *SPP1* were significantly associated with milk protein percentage (15). Therefore, further studies on the effects of *SPP1* on milk production traits in dairy cows are needed to elucidate the molecular mechanisms of QTL effects (14).

*NCAPG* is a subunit of Condensin I located on bovine chromosome 6 (16). It plays an essential role in chromatin compression and chromosome segregation during mitosis and meiosis (17). GWAS identified *NCAPG* as being associated with muscle growth and development (5). In skeletal muscle myoblasts from Holstein cows, knockdown of *NCAPG* promotes apoptosis, prolongs mitosis, and impairs the process of myogenic differentiation in fetal bovine myoblasts (16). It can be inferred that the regulatory processes of *NCAPG* in organisms are highly complex and diverse. It has been found that the proliferation and apoptosis of mammary epithelial cells during lactation affect lactation (18). However, no studies have been conducted regarding the role of *NCAPG* on milk production traits. Therefore, it is necessary to carry out polymorphism studies on *NCAPG*.

The objectives of this study were to characterize genetic variation in the *SPP1* and *NCAPG* genes in Holstein cows and to test the association of these SNPs with milk production traits in Holstein cow populations. The effects of *SPP1* and *NCAPG* on casein and triglyceride synthesis as well as the regulation of cell proliferation and apoptosis in bovine mammary epithelial cells (BMECs) were further explored to provide reference information for in-depth mechanistic studies of candidate genes related to milk production traits in dairy cows.

## Materials and methods

### Animal selection and phenotypic data collection

In this study, a total of 1,114 Holstein cows were involved in a study population of Holstein cows in Ningxia, selected from Helan Mountain Dairy Co. The selected test population had accurate DHI determination records, and individual information (cow number, birth date, litter size, day old, etc.), where the herd birth dates are clustered from 2014 to 2021. Five milk production traits were mainly analyzed: 305 days milk yield, 305 days of milk fat, milk fat percentage, 305-days milk protein yield, and milk protein percentage. The data were all provided by the DHI measurement laboratory of Ningxia Animal Husbandry Station.

### Enrichment analysis

Omicshare online software was used for gene ontology (GO) annotation to analyze the annotation function of genes. Pathway analysis of the identified milk proteins was performed based on the online Omicshare software using the Kyoto Encyclopedia of Genes and Genomes (KEGG) pathway database. The results of GO and KEGG enrichment of candidate genes obtained based on the previous GWAS screening are shown in Supplementary Tables S1, S2. Pearson correlations were calculated for each class of traits using CorrPlot and plotted using the OmicStudio tool at https://www.omicstudio.cn/tool.

### DNA extraction, SNP identification, and genotyping

Blood was collected from the tail root of a selected group of heifers and blood DNA was extracted from Holstein cows using a DNA extraction kit and a Nanodrop 2000 spectrophotometer (Thermo Fisher Scientific, United States), and a 1.5% agarose gel was used to determine the concentration and quality of the extracted DNA. Fifty DNA samples that passed the test were randomly selected, all diluted to a concentration of 50 ng/μL, and mixed in equal amounts to construct a DNA mixing pool, and the DNA from the mixing pool of these 50 Holstein cattle was used as a template. Based on the sequences of *NCAPG* (NM_001102376.2) and *SPP1* (NM_174187.2) genes in NCBI^1^, primers were designed for *SPP1* and *NCAPG* genes using Primer 5.0 and synthesized by Shaanxi Prime Biotech (Supplementary Table S3). After PCR amplification (Supplementary Table S4), the PCR products were detected by 1% agarose gel electrophoresis and sequenced by Shaanxi Prime Biological Company. Sequencing maps and sequences were analyzed using SnapGene7.2.0 software, and SNP locations and mutation types were identified by comparison. According to the type of base mutation site to be typed and sequence characteristics, MassARRAY mass spectrometry was selected for detection in this study.

### Linkage disequilibrium estimation association analyses

Gene frequency, genotype frequency, genetic heterozygosity (He), number of effective alleles (Ne), polymorphic information content (PIC) and chi-square test value χ2 were calculated for each SNP locus allele, and haplotype linkage disequilibrium analysis was performed at the SNP loci using Haploview 4.2 software. Association analyses were performed using the MIXED program of SAS 9.4 to analyze associations between *SPP1* and *NCAPG* polymorphisms and milk production traits using the following mixed linear model: Y = *μ* + YS + P + M + G + a + e, where Y is the phenotypic value of each trait for each cow; μ is the overall mean; YS is the year (1–4, 2017–2021) and season (1, March–May, 2, June–August, 3: September–November; 4: December–February); P is the litter effect (1–7); M is the age at calving as a covariate; and G is the genotype or haplotype group and effect; a is the individual random additive genetic effect, distributed as N (0, Aδ2 a), with the additive genetic variance δ2 a; and e is the random residual, distributed as N (0, Iδ2 e), with identity matrix I and residual error variance δ2 e. Differences are significant when *p* < 0.05 and highly significant when *p* < 0.01.

### Cell culture and transfection

Tissues and cells used in this experiment were pre-preserved by our research group. BMECs were cultured in Dulbecco’s modified Eagle medium (DMEM)/F12 (BI, Jerusalem, Israel) supplemented with 10% fetal bovine serum (FBS) (CellMax, Beijing, China) and 1% penicillin/streptomycin (Solarbio, Beijing, China) at 37°C with 5% CO2. Three complementary pairs of siRNA and si-NC of *SPP1* and *NCAPG* genes were synthesized in Gemma Bio respectively, the specific synthesized sequences are shown in Supplementary Table S5. where si-NC was the negative control group. The synthesized siRNAs of *SPP1* and *NCAPG* genes were transfected into BMECs using ZETA LIFE transfection reagent according to the reagent instructions, respectively, and after 72 h, cell cultures and cells were collected for further analysis.

### RNA extraction and quantitative real-time PCR

Total RNA from tissues and BMECs was extracted using RNAiso reagent (Takara, Dalian, China). The cDNA was synthesized via reverse transcription following the instructions provided by the PrimeScript RT Reagent Kit (Perfect Real Time) (Takara, Dalian, China). For mRNA primers were designed with Primer Premier 6. GAPDH served as the internal reference for mRNA, and quantitative PCR was conducted on a CFX96 detection system (Bio-Rad) using the SYBR Green Premix Pro Taq HS qPCR Kit (Accurate Biotechnology (Hunan) Co., Ltd., Changsha, China). The relative expression levels of mRNA were analyzed employing a 2^−ΔΔCt^ model. Primer information for qPCR is detailed in Supplementary Table S6.

### Cell viability and proliferation

For the CCK-8 assay, the BMECs were seeded in 96-well plates and transfected when the cell adhered to the wall. Cell viability was detected every 12 h after transfection and calculated by measuring absorbance at 450 nm using SuPerMax 3,200 according to the manufacturer’s protocols of the CCK-8 kit (Beyotime, Shanghai, China).

The expression of the cell proliferation marker gene was detected by transfection of interfering small RNA at 48 h. Cell proliferation assay kit (Beyotime, Shanghai, China) was used and incubated with 1 × EDU working solution for 2 h at 37°C. Cells were then fixed with 4% paraformaldehyde for 15 min at room temperature and permeabilized with 0.5% Triton X-100 for 15 min before BMECs were incubated with 200 μL of Click Additive Solution for 30 min at room temperature. Subsequently, the cells were incubated with 1× Hoechst solution at room temperature away from light for 10 min. A fluorescence-inverted microscope was used to capture images of EDU. The percentage of EDU-labeled positive cells was analyzed using Image, proliferating cells labeled with EDU showed bright red fluorescence under a fluorescence microscope, and nuclei were stained by DAPI, which showed bright blue color, and the percentage of cell proliferation was detected using the number of EDU-labeled cells over the number of nuclei of DAPI-labeled cells in each group.

### Cell apoptosis assays

The expression of the apoptosis marker gene was detected by transfection of interfering small RNA at 48 h. At the same time, apoptosis was detected by Annexin V-mCherry/SYTOX Green apoptosis detection kit (Beyotime, Shanghai, China) at 48 h. According to the manufacturer’s instructions, BMECs were digested and collected with trypsin (Hyclone, United States). After centrifugation, 194 μL Annexin V-mCherry Binding Buffer resuspension cells were added, and then 5 μL Annexin V-mCherry and 1 μL SYTOX Green were added and incubated at room temperature for 20 min. After incubation, the cells were collected by centrifugation and resuscitated with 50 μL Annexin V-mCherry Binding Buffer. After the smear, the cells were observed under the fluorescence microscope. The nuclei of apoptotic cells were labeled with green fluorescence by SYTOX Green fluorescent dye, and apoptotic and necrotic cell membranes were labeled with red fluorescence by Annexin V-mCherry fluorescent dye.

### Determination of triglyceride content

BMECs were collected 48 h after transfection, and the triglyceride content was determined using a cell-specific high-fat sample triglyceride (TG) enzymatic assay kit (Applygen, Beijing, China). According to the reagent instructions, the lysed supernatant was added to the prepared working solution, and the reaction was performed at 37°C for 15 min, and the OD value of each tube was detected at 550 nm out, and the TG content was corrected by the concentration of protein per mg.

### Casein content test

In this experiment, ELISA was used to determine the content of *β*-casein, k-casein and αs1-casein secreted by BMECs. The assay was performed according to the instructions provided in the kit (Meimian, Jiangsu, China). After 72 h of transfection, the cell culture was collected in a 1.5 mL centrifuge tube and centrifuged at 3000 r/min for 20 min, and the supernatant was aspirated as the sample to be tested. The plate was equilibrated at room temperature for 20 min before use, and 50 ul of different concentrations of standards were added to the standard wells, while 10 μL of the samples to be tested were added to the sample wells, followed by 40 μL of the sample dilution, and three replicates were set up for each sample well. After that, horseradish peroxidase-labeled detection antibody, substrate A, substrate B and termination solution were added according to the instructions, and then the absorbance values of each well were measured at 450 nm.

### Statistical analysis

Data analysis and visualization were completed using GraphPad Prism (version 8.0) software. Data are presented as the mean ± SEM. The difference between the two groups was compared using a two-tailed Student’s *t*-test and comparisons among multiple groups were performed with a one-way analysis of variance followed by Dunnett’s test. *p* > 0.05 is considered not significant, *p* < 0.05 for significant difference and *p* < 0.01 for highly significant difference (ns represents *P*>0.05,* represents *p* < 0.05 and ** represents *p* < 0.01). All experiments were conducted at least three times.

## Results

### Functional annotation and interplay analysis of *SPP1* and *NCAPG* genes

**Figure 1:**
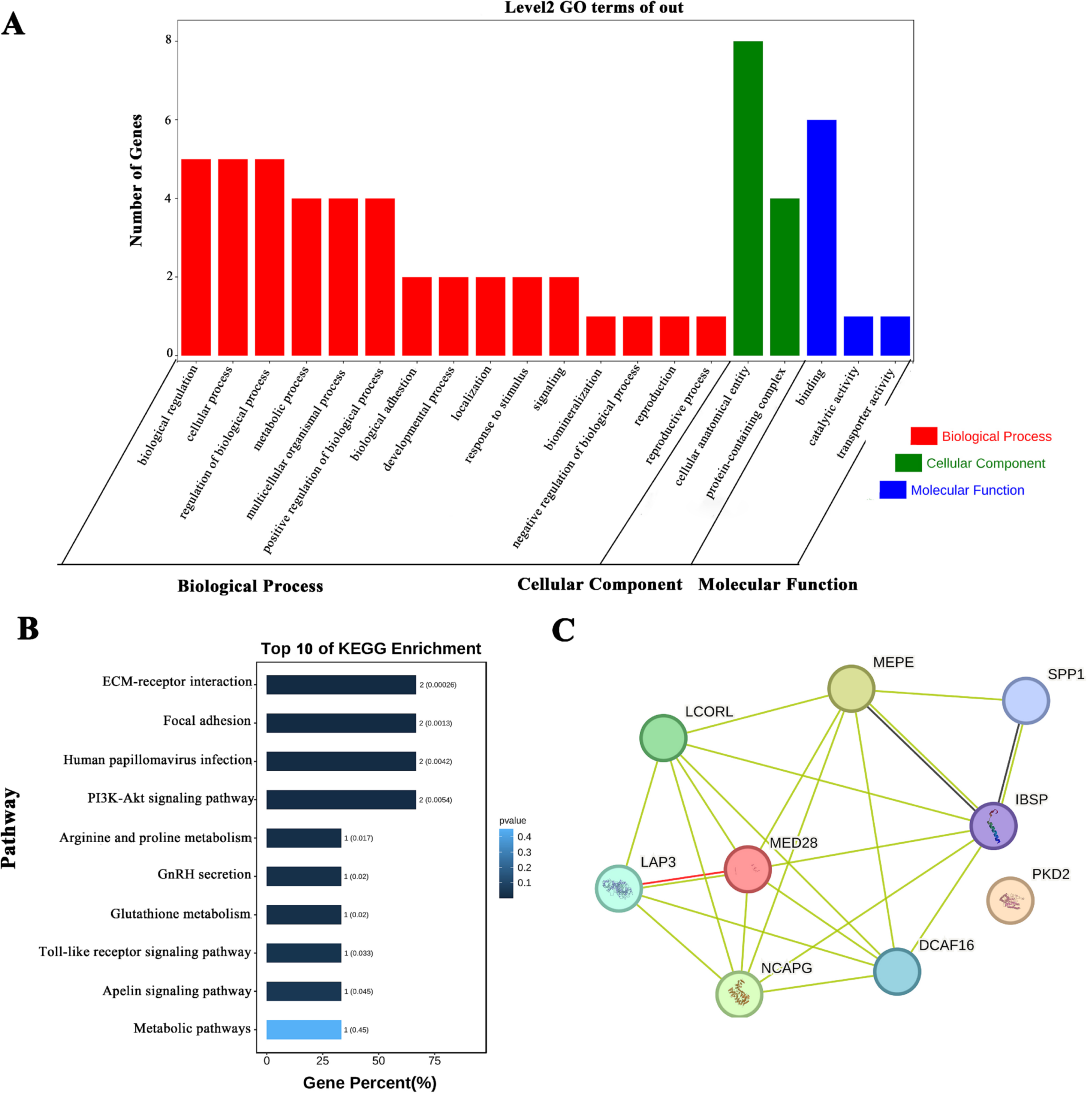
*SPP1* and *NCAPG* enrichment analysis and protein interactions. **(A)** GO analysis of milk production trait candidate genes; **(B)** KEGG analysis of the first 10 milk production trait candidate genes; **(C)** protein interaction analysis of milk production trait candidate genes.

Based on the GWAS results, many QTLs related to milk production traits and SCS existed on chromosome 6 in cattle, and we performed GO and KEGG enrichment analyses for the pre-screened *PKD2*, *SPP1*, *MEPE*, *IBSP*, *LAP3*, *MED28*, *DCAF16*, *NCAPG*, and *LCORL* genes. The GO results showed that (Figure 1A). Both *SPP1* and *NCAPG* were enriched in biological process (BP), cellular component (CC) and molecular function (MF). The BP analysis results showed that *SPP1* and *NCAPG* were mainly enriched regulation of biological process and cellular processes. The MF categorization showed that *SPP1* was involved in binding. The CC results indicated that involved in cellular and protein composition. KEGG results showed (Figure 1B) that *SPP1* was enriched in pathways mainly PI3K-Akt signaling pathway, GnRH secretion and Toll-like receptor signaling pathway, etc. STRING results showed that all genes, except *PDK1*, interacted with each other (Figure 1C).

### Screening of SNP mutation sites in the *SPP1* and *NCAPG* genes

**Figure 2:**
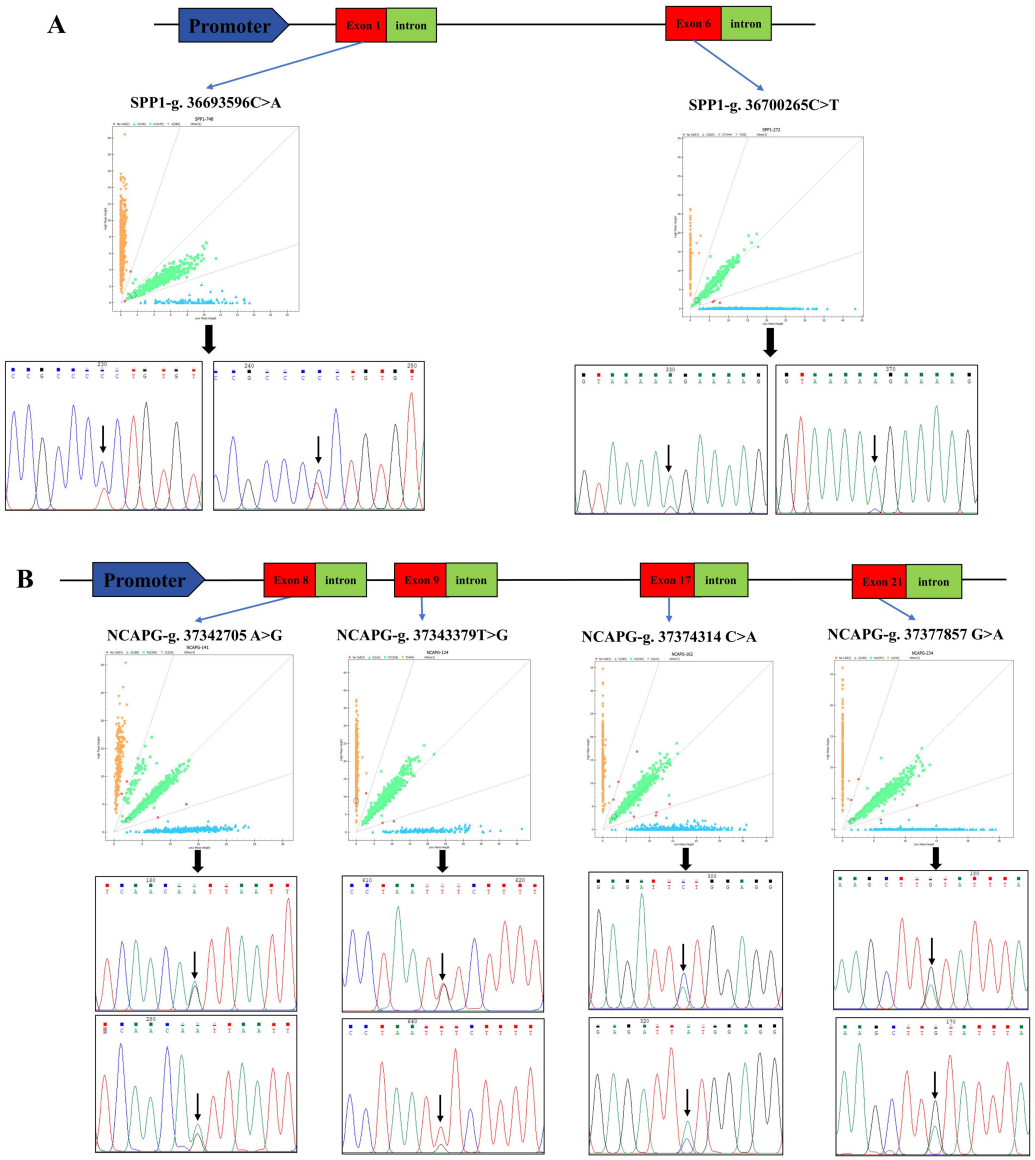
Identification of *SPP1* and *NCAPG* SNPs. **(A)** Schematic diagram of *SPP1* genome structure, genotyping scatter plot and site-specific sequencing peaks, red squares represent exon regions, green squares represent intron regions, the different colored lines represent different bases, and the black arrows point to bases that are mutated. **(B)** Schematic diagram of *NCAPG* genome structure, genotyping scatter plot and site-specific sequencing peaks, red squares represent exon regions, green squares represent intron regions, and different colored lines represent different bases. Red squares represent exon regions, green squares represent intron regions, the different colored lines represent different bases, and the black arrows point to bases that are mutated.

The sequencing results were compared with the sequences of *SPP1* and *NCAPG*, and two SNPs and four SNPs were found in *SPP1* and *NCAPG*, respectively: *SPP1*-g. 36,693,596 C > A, *SPP1*-g. 36,700,265 C > T (Figure 2A), *NCAPG*-g. 37,343,379 T > G, *NCAPG*-g. 37,377,857 G > A, *NCAPG*-g. 37,374,314 C > A, *NCAPG*-g. 37,342,705 A > G (Figure 2B). All loci are located within the exonic region. The *SPP1* gene has three genotypes per SNP locus. The dominant genotypes for *SPP1*-g. 36,700,265 C > T and *SPP1*-g. 36,693,596 C > A are CC and AA. The *NCAPG* gene has three genotypes per SNP locus. The *NCAPG*-g. 37,342,705 C > A, *NCAPG*-g. 37,343,379 G > A,*NCAPG*-g. 37,342,705 C > A, *NCAPG*-g. 37,343,379 G > A,*NCAPG*-g. 37,343,379 G > A, and *NCAPG*-g. 37,343,379 G > A have the following genotypes. g. 37,343,379 G > T, *NCAPG*-g. 37,374,314 C > A and *NCAPG*-g. 37,377,857 G > A with dominant genotypes CA, TG, CA, and GA. The genetic diversity of these six SNPs was investigated by estimating the genotype frequency, allele frequency, polymorphism information content (PIC) and Hardy Weinberg equilibrium values (Table 1). All SNPs were in Hardy–Weinberg equilibrium (*p* > 0.05).

**Table 1:** Genetic information of six SNPs identified by *SPP1* and *NCAPG.*

SNPs	Location	Number	Genotype	Genotype frequency	Allele	Allele number	Allele frequency	χ^2^HWE	PIC	He	Ne
*SPP1*-g. 36,700,265 C > T	Exon 1	82	TT	0.071	C	583	0.733	0.525	0.31	0.39	1.62
		419	CT	0.392	T	1,599	0.267				
		590	CC	0.537							
*SPP1*-g. 36,693,596 C > A	Exon 6	101	CC	0.089	C	651	0.702	0.557	0.33	0.42	1.70
		449	CA	0.418	A	1,535	0.298				
		543	AA	0.493							
*NCAPG*-g. 37,342,705 A > G	Exon 8	215	CC	0.196	C	963	0.443	0.721	0.37	0.49	1.9
		530	AG	0.493	A	1,213	0.557				
		341	AA	0.311							
*NCAPG*-g. 37,343,379 G > T	Exon 9	153	GG	0.139	G	811	0.373	0.822	0.36	0.47	1.87
		504	TG	0.468	T	1,363	0.627				
		429	TT	0.393							
*NCAPG*-g. 37,374,314 C > A	Exon 17	216	AA	0.195	A	958	0.441	0.577	0.37	0.49	1.94
		527	CA	0.493	C	1,212	0.559				
		343	CC	0.312							
*NCAPG*-g. 37,377,857 G > A	Exon 21	216	AA	0.196	A	964	0.443	0.653	0.37	0.49	1.95
		529	GA	0.493	G	1,214	0.557				
		341	GG	0.311							

^abc^The different letters stand for significant differences (*p* < 0.05), ^ABC^The different letters stand for highly significant differences (*p* < 0.01), while the same letter indicates no difference (*p* > 0.05).

### Correlation analysis of milk production traits in Holstein cows

**Figure 3:**
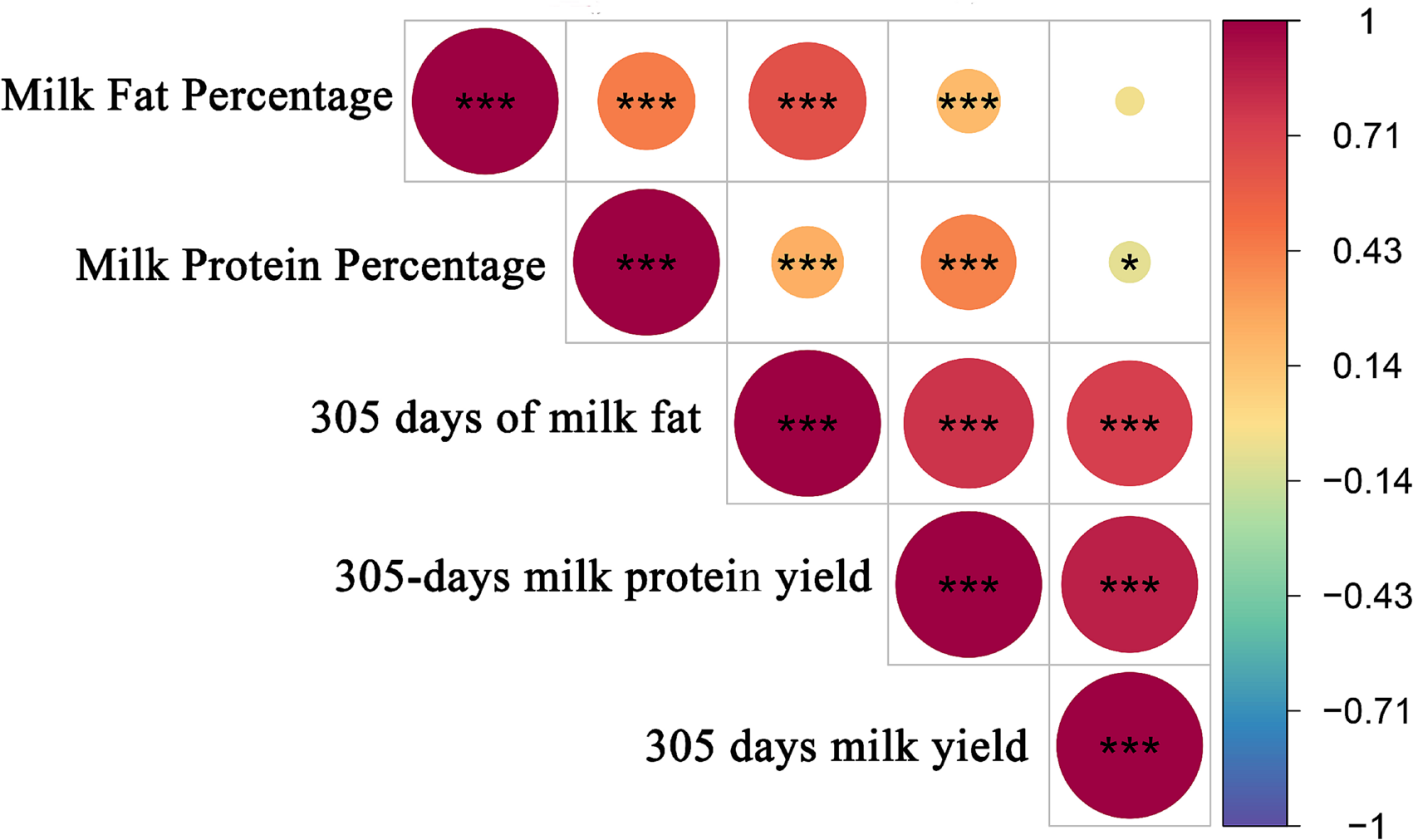
Pearson correlation analysis of milk production traits. The results are plots of correlation coefficients for milk production traits. *** and** in the graph represents correlation *p* < 0.01, and * in the graph represents correlation *p* < 0.05.

To further understand the information about the selected Holstein cow population, Pearson correlation coefficients were calculated for these five milk production traits. The correlations of milk production traits in Holstein cows are shown in Figure 3. There were highly significant positive correlations (*p* < 0.01) between milk fat percentage and milk protein percentage and 305 days of milk fat and 305-days milk protein yield, and significant negative correlations (*p* < 0.05) between milk protein percentage and 305 days milk yield. There was a highly significant positive correlation between milk fat percentage and milk protein percentage (*p* < 0.01).

### Association analysis of *SPP1* and *NCAPG* gene genotypes with milk production traits

The different genotypes of SNPs in the *SPP1* and *NCAPG* genes were analyzed for association with Milk Fat Percentage, Milk Protein Percentage, 305 days of milk fat, 305-days milk protein yield, and 305 days milk yield in Holstein cows (Table 2). The results showed that *SPP1*-g. 36,700,265 C > T significantly affected Milk Protein Percentage (*p* < 0.01), in which TT genotypes of cows had higher milk protein percentages than CC and CT genotypes. *SPP1*-g. 36,693,596 C > A significantly affected Milk Protein Percentage (*p* < 0.05), in which CC genotypes of cows had higher milk protein percentage than AA and CA genotypes. Had significantly higher milk protein rate than AA and CA genotypes. *NCAPG*-g. 37,342,705 C > A significantly affected milk protein percentage (*p* < 0.05), where AA genotypes of cows had significantly higher milk protein percentage than CC and CA genotypes (*p* < 0.05). *NCAPG*-g. 37,343,379 G > T significantly affected milk protein percentage (*p* < 0.05), where TT and TG genotypes had significantly higher milk protein percentage than GG genotypes (*p* < 0.05). *NCAPG*-g. 37,374,314 C > A significantly affected milk protein percentage (*p* < 0.05), with CC genotypes significantly higher than AA and CA genotypes (*p* < 0.05). *NCAPG*-g. 37,377,857 G > A significantly affected milk protein percentage (*p* < 0.05), with GG genotypes significantly higher than AA and GG genotypes (*p* < 0.05). milk protein percentage was significantly higher (*p* < 0.05) than AA and GA genotypes.

**Table 2:** Effect of different genotypes of SNPs of the *SPP1* and *NCAPG* gene on milk production traits in Holstein cows.

SNP locus	Genotype	Record number	Milk fat percentage (%)	Milk Protein Percentage (%)	305 days of milk fat (kg)	305 days milk protein yield (kg)	305 days milk yield (kg)
*SPP1*-g. 36,700,265 C > T	CC	590	4.00 ± 0.03	3.30 ± 0.01^B^	470.76 ± 5.05	387.75 ± 3.46	11777.11 ± 94.86
	TT	82	4.01 ± 0.09	3.43 ± 0.04^A^	477.57 ± 15.46	406.95 ± 10.15	11893.00 ± 281.38
	CT	419	4.04 ± 0.04	3.34 ± 0.02^B^	465.50 ± 6.23	385.01 ± 4.48	11550.91 ± 125.41
	*P*		0.686	0.005	0.666	0.116	0.260
*SPP1*-g. 36,693,596 C > A	AA	543	4.01 ± 0.03	3.30 ± 0.02^b^	473.21 ± 5.19	389.23 ± 3.59	11809.45 ± 97.64
	CC	101	4.06 ± 0.08	3.42 ± 0.04^a^	481.21 ± 13.74	404.26 ± 8.86	11853.35 ± 249.78
	CA	447	4.01 ± 0.03	3.33 ± 0.02^b^	461.74 ± 6.08	383.16 ± 4.35	11529.83 ± 121.97
	*P*		0.822	0.010	0.219	0.085	0.157
*NCAPG*-g. 37,342,705 C > A	AA	341	4.05 ± 0.04	3.37 ± 0.02^a^	473.70 ± 6.55	395.43 ± 4.87	11765.11 ± 136.22
	CC	215	3.93 ± 0.05	3.29 ± 0.02^b^	468.38 ± 8.75	391.57 ± 5.71	11920.45 ± 154.54
	CA	530	4.04 ± 0.03	3.31 ± 0.02^b^	466.71 ± 5.56	381.83 ± 3.81	11553.79 ± 105.30
	*P*		0.073	0.016	0.722	0.067	0.139
*NCAPG*-g. 37,343,379 G > T	GG	153	3.90 ± 0.06	3.28 ± 0.03^b^	465.47 ± 10.50	389.30 ± 6.87	11866.90 ± 179.01
	TT	429	4.05 ± 0.03	3.36 ± 0.02^a^	473.41 ± 5.97	393.10 ± 4.28	11740.97 ± 108.68
	TG	504	4.02 ± 0.03	3.31 ± 0.02^a^	466.74 ± 5.64	383.37 ± 3.91	11600.77 ± 108.68
	*P*		0.096	0.036	0.669	0.236	0.432
*NCAPG*-g. 37,374,314 C > A	AA	216	3.92 ± 0.05	3.29 ± 0.02^b^	469.19 ± 8.74	391.91 ± 5.70	11923.58 ± 154.41
	CC	343	4.05 ± 0.04	3.37 ± 0.02^a^	473.72 ± 6.52	395.55 ± 4.84	11767.20 ± 154.41
	CA	527	4.04 ± 0.03	3.31 ± 0.02^b^	466.77 ± 5.58	381.92 ± 3.83	11557.66 ± 105.83
	*P*		0.097	0.016	0.729	0.064	0.141
*NCAPG*-g. 37,377,857 G > A	AA	216	3.92 ± 0.05	3.29 ± 0.02^b^	468.58 ± 8.72	391.47 ± 5.69	11915.65 ± 153.90
	GG	341	4.04 ± 0.04	3.37 ± 0.02^a^	473.38 ± 6.53	395.55 ± 4.85	11773.24 ± 136.06
	GA	529	4.04 ± 0.03	3.31 ± 0.02^b^	466.63 ± 5.57	381.86 ± 3.82	11555.06 ± 105.49
	*P*		0.094	0.018	0.740	0.065	0.142

^abc^The different letters stand for significant differences (*p* < 0.05), ^ABC^The different letters stand for highly significant differences (*p* < 0.01), while the same letter indicates no difference (*p* > 0.05).

### Linkage disequilibrium and haplotype analysis

The two SNPs detected by *SPP1* and four SNPs detected by *NCAPG* were analyzed for linkage disequilibrium and haplotype using Haploview 4.2 software, with one haplotype module was identified for each, respectively.

**Figure 4:**
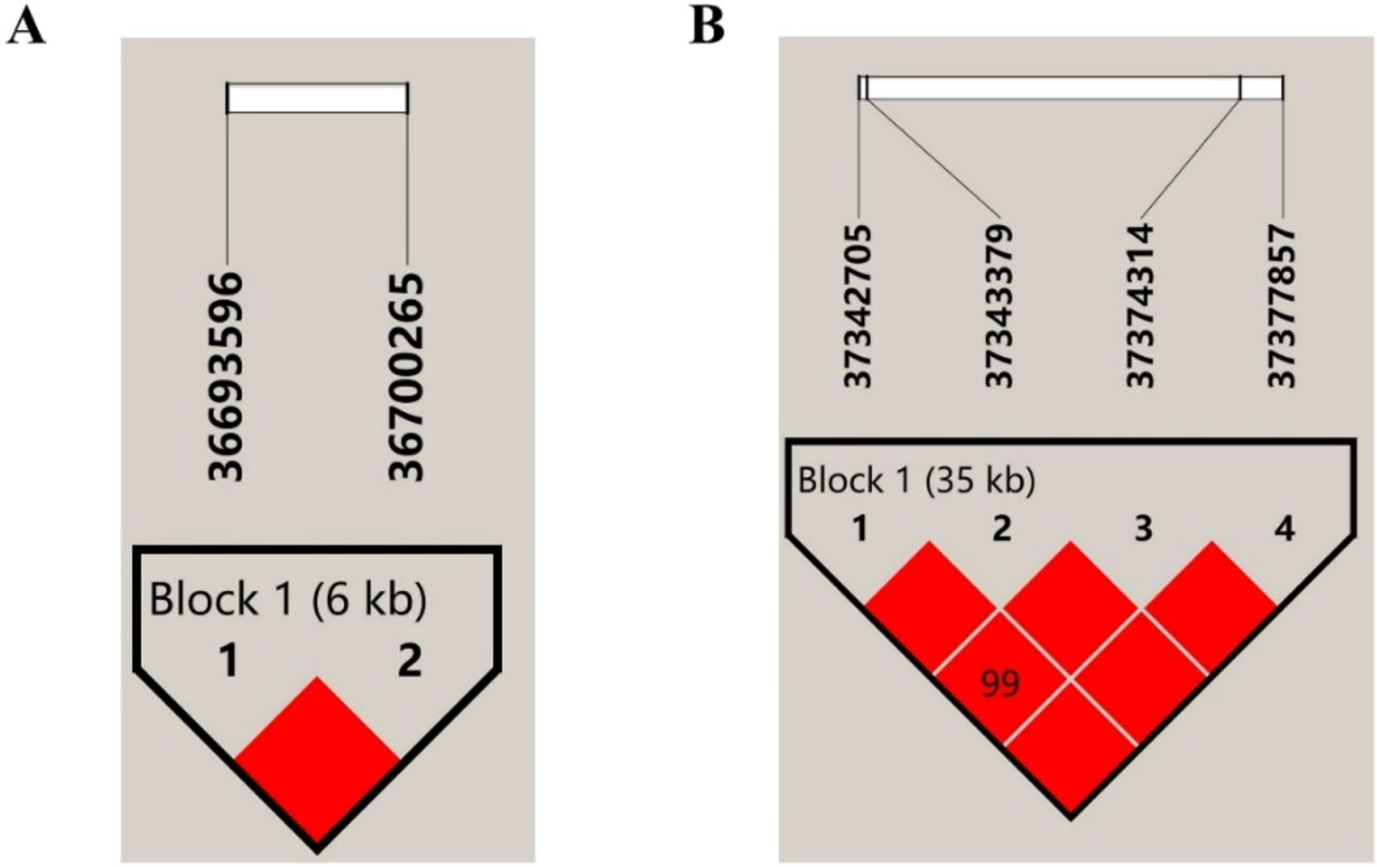
Linkage disequilibrium of SNPs in *SPP1* and *NCAPG.* **(A)** LD estimation between 2 SNPs in *SPP1*, Block 1 (6 kb) indicates a haplotype block, the text above the horizontal number is the SNP name, and the cross value of the SNPs indicates the degree of LD D′, and the red square without a numerical value indicates that the 2 SNPs are completely interlocked (D′ = 1); **(B)** LD estimation among 4 SNPs in *NCAPG*, Block 1 (35 kb) indicates haplotype block, the text above the horizontal number is the SNP name, the cross value of SNPs indicates the degree of LD D′, and the red squares without values indicate that 2 SNPs are fully interlocked (D′ = 1).

This haplotype block of *SPP1* is composed of *SPP1*-g. 36,700,265 C > T and *SPP1*-g. 36,693,596 C > A constitute a structural domain with a length of about 6 kb, D′ = 1, both with extremely strong LD levels, and AC, CT, and CC as its three major haplotypes, with a frequency of 70.3, 26.7, and 3%, respectively (Figure 4A). The results of correlation analysis of these three haplotypes were shown in Table 3, which showed that the milk protein percentage of haplotype H2H2 was very significantly higher than that of H1H1, H1H2, H1H3, and H3H3 (*p* < 0.01), and the different combinations of haplotypes did not have any significant effect on Milk Fat Percentage, 305 days of milk fat, 305-days milk protein yield, and 305 days milk yield. Milk Fat Percentage, 305 days of milk fat, 305-days milk protein yield and 305 days of milk yield were not significantly affected by different haplotype combinations (*P*>0.05). The combined analysis of the dominant haplotype was H2H2, i.e., CCTT. under the dominant haplotype, *SPP1*-g. 36,693,596 C > A was the pure haplotype CC and *SPP1*-g. 36,700,265 C > T was the pure haplotype TT, which was consistent with the results of the single-marker association analysis.

**Table 3:** Analysis of the correlation between *SPP1* gene haplotypes and milk production traits in Chinese Holstein cows.

Haplotypes	Milk fat percentage (%)	Milk protein percentage (%)	305 days of milk fat (kg)	305 days milk protein yield (kg)	305 days of milk yield (kg)
H1H1	4.01 ± 0.03	3.32 ± 0.01^B^	474.46 ± 123.77	391.59 ± 84.57	11816.34 ± 2311.61
H1H2	4.02 ± 0.02	3.34 ± 0.01^B^	463.56 ± 128.07	384.05 ± 91.86	11540.03 ± 2571.64
H1H3	4.00 ± 0.22	3.31 ± 0.01^B^	466.87 ± 125.32	385.77 ± 87.58	11670.52 ± 2428.22
H2H2	4.04 ± 0.59	3.42 ± 0.03^A^	479.59 ± 138.58	405.46 ± 90.11	11871.11 ± 2520.36
H2H3	4.04 ± 0.32	3.36 ± 0.02^AB^	468.55 ± 129.71	388.75 ± 91.47	11609.66 ± 2556.55
H3H3	4.01 ± 0.27	3.31 ± 0.14^B^	472.29 ± 125.03	390.16 ± 84.94	11788.25 ± 2333.67
*p* value	0.931	0.002	0.438	0.052	0.174

H1 = AC, H2 = CT, H3 = CC. ^abc^The different letters stand for significant differences (*p* < 0.05), ^ABC^The different letters stand for highly significant differences (*p* < 0.01), while the same letter indicates no difference (*p* > 0.05).

The haplotype block *NCAPG* is composed of six structural domains consisting of *NCAPG*-g. 37,342,705 C > A, *NCAPG*-g. 37,343,379 G > T, *NCAPG*-g. 37,374,314 C > A, and *NCAPG*-g. 37,377,857 G > A, and there is a very strong LD level between these four SNPs (D’ = 0.99 ~ 1), and a haplotype block was constructed from them with a length of about 35 kb, with ATCG, GGAA and GTAA as the main three haplotypes with frequencies of 56, 37 and 7%, respectively (Figure 4B). The three haplotypes were analyzed for association, and the results were shown in Table 4, which showed that the haplotypes were significantly correlated with the milk protein percentage (*p* < 0.05) and highly significantly correlated with the association of 305-days milk protein yield and 305 days milk yield (*p* < 0.01).

**Table 4:** Analysis of the correlation between *NCAPG* gene haplotypes and milk production traits in Chinese Holstein cows.

Haplotypes	Milk fat percentage (%)	Milk protein percentage (%)	305 days of milk fat (kg)	305 days milk protein yield (kg)	305 days milk yield (kg)
H1H1	4.00 ± 0.02	3.34 ± 0.01^ab^	471.73 ± 3.59	393.23 ± 2.51^AC^	11812.07 ± 69.53^A^
H1H2	4.04 ± 0.16	3.31 ± 0.01^b^	466.71 ± 2.79	382.23 ± 1.92^B^	11566.42 ± 53.11^B^
H1H3	4.04 ± 0.16	3.32 ± 0.01^bc^	468.13 ± 2.83	384.26 ± 1.96^BC^	11594.97 ± 54.32^BC^
H2H2	4.00 ± 0.22	3.34 ± 0.01^ac^	471.48 ± 3.81	393.85 ± 2.71^A^	11815.10 ± 74.69^A^
H2H3	4.04 ± 0.19	3.34 ± 0.01^a^	470.43 ± 3.31	390.58 ± 2.36^ABC^	11732.36 ± 65.46^AC^
H3H3	4.03 ± 0.19	3.35 ± 0.01^a^	472.79 ± 3.37	394.14 ± 2.43^A^	11785.18 ± 25.57^A^
*p* value	0.492	0.016	0.718	0.000	0.005

H1 = ATCG, H2 = GGAA, H3 = GTAA. ^abc^The different letters stand for significant differences (*p* < 0.05), ^ABC^The different letters stand for highly significant differences (*p* < 0.01), while the same letter indicates no difference (*p* > 0.05).

### Tissue expression profile and interference efficiency assay of *SPP1* and *NCAPG*

**Figure 5:**
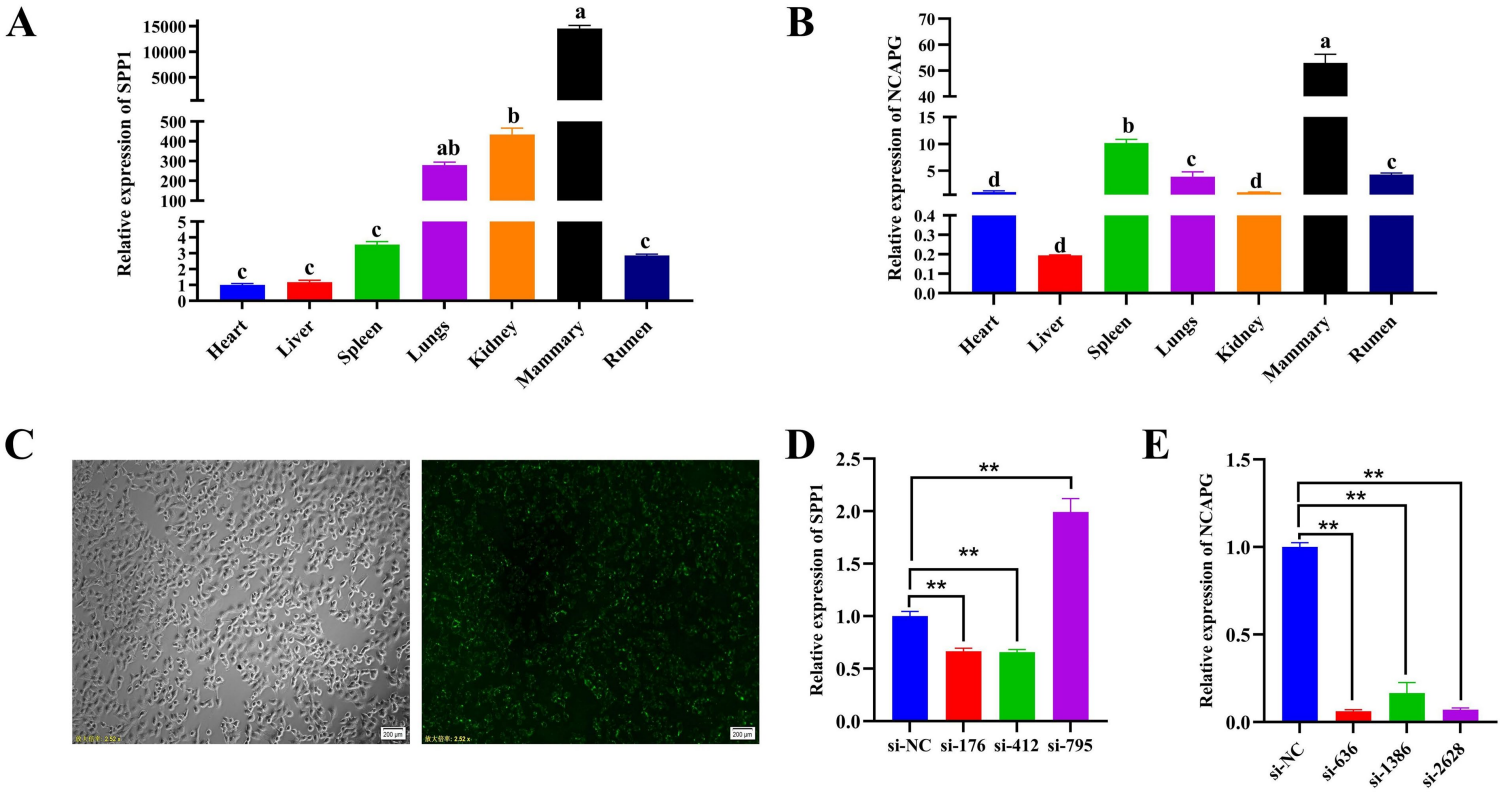
Tissue expression profile and interference efficiency assay of *SPP1* and *NCAPG.* **(A)** qRT-PCR to detect the expression level of *SPP1* in different tissues of dairy cows; **(B)** qRT-PCR to detect the expression level of *NCAPG* in different tissues of dairy cows; **(C)** FAM-labeled si-NC bright-field field of view (100 ×) and FAM-labeled si-NC transfected field of view (100 ×); **(D)** qRT-PCR to detect the *SPP1* interference fragment efficiency; **(E)** qRT-PCR to detect *NCAPG* interference fragment efficiency. Differences between two groups were compared using a two-tailed Student’s *t*-test, and comparisons between multiple groups were analyzed by one-way ANOVA. ** Indicates highly significant differences (*p* < 0.01) and * indicates significant differences (*p* < 0.05).

Both *SPP1* and *NCAPG* were expressed at different levels in various tissues of dairy cows. *SPP1* had the highest expression level at the mammary level and the lowest in the heart (Figure 5A). *NCAPG* had the highest expression level in the mammary tissues of dairy cows and the lowest in the liver (Figure 5B). To further reveal the biological functions of *SPP1* and *NCAPG* in BMECs, three different siRNAs for *SPP1*: si-176, si-412, and si-795; and three different siRNAs for *NCAPG*: si-636, si-1386, and si-2628, were designed and synthesized. siRNAs were synthesized into siRNAs, si-NC, and FAM-labeled si-NC were transfected into BMECs respectively, and after 9 h of transfection, a fluorescence inverted microscope was used to observe the transfection, and the results showed that the transfection was successful (Figure 5C). qRT-PCR results showed that the transfection of si-412 from *SPP1* and si-636 from *NCAPG* had the best interference efficiency. si-412 had an interference efficiency of 28.4% (Figure 5D), and the interference efficiency of si-636 amounted to 93.9% (Figure 5E). Therefore, si-412 and si-636 were selected as siRNAs for subsequent experiments.

### Effect of *SPP1* on proliferation and apoptosis of BMECs

**Figure 6:**
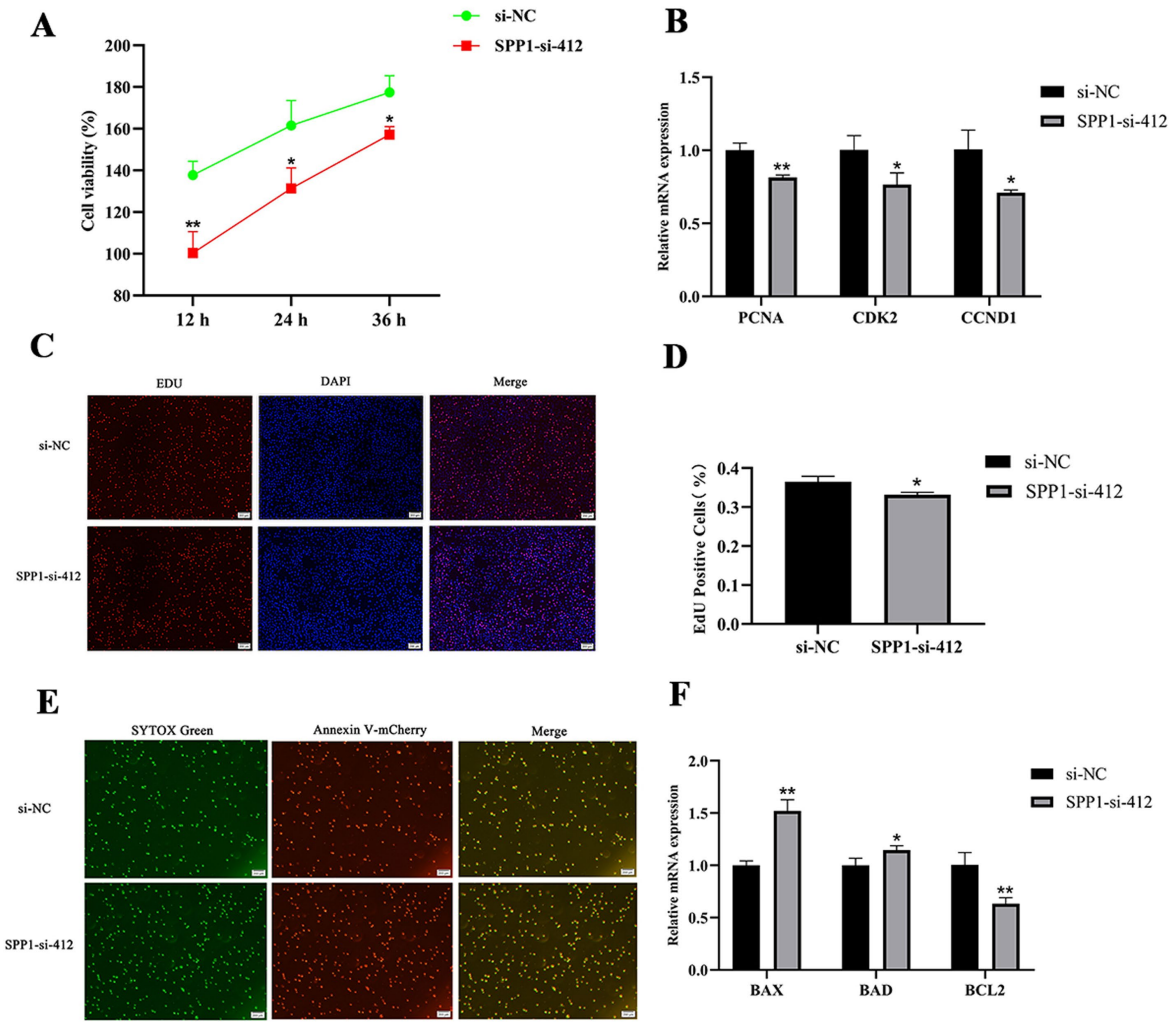
Effect of *SPP1* on proliferation and apoptosis of BMECs. **(A)** Cell viability was detected by CCK8 after interfering with *SPP1*; **(B)** the expression level of proliferation marker genes was detected after interfering with *SPP1*; **(C,D)** cell proliferation was detected by EDU after interfering with *SPP1* for 48 h and the percentage of EDU-labeled positive BMECs was counted by Image J software; **(E)** cell apoptosis was detected after interfering with *SPP1* for 48 h using Annexin V- mCherry/SYTOX Green to detect apoptosis; **(F)** the expression levels of apoptosis marker genes were detected after interference with *SPP1*. Differences between two groups were compared using a two-tailed Student’s *t*-test, and comparisons between multiple groups were analyzed by one-way ANOVA. ** Indicates highly significant differences (*p* < 0.01) and * indicates significant differences (*p* < 0.05).

To explore the effects of *SPP1* on the proliferation and apoptosis of BMECs. CCK8 results showed (Figure 6A) that *SPP1*-si-412 highly significantly inhibited cell viability at 12 h (*p* < 0.01) and significantly inhibited cell viability at 24 h and 24 h (*p* < 0.05). qRT-PCR results showed that compared with si-NC, *SPP1*-si-412 highly significantly inhibited the expression of PCNA (*p* < 0.01) and significantly inhibited the expression of *CDK2* and *CCND1* (*p* < 0.05) (Figure 6B); moreover, EDU results showed that *SPP1*-si-412 could significantly inhibit the percentage of positive cell proliferation compared with si-NC (*p* < 0.05) (Figures 6C,D). The results of the apoptosis assay showed that the number of apoptotic BMECs was significantly increased after knockdown of *SPP1* (Figure 6E), and *SPP1*-si-412 could highly significantly up-regulate the expression level of *BAX* (*p* < 0.01), significantly up-regulate the expression level of *BAD* (*p* < 0.05), and highly significantly inhibit the expression of the anti-apoptotic gene *BCL2*, compared with si-NC (*p* < 0.01) (Figure 6F). In summary, interference with *SPP1* inhibited the proliferation and viability of BMECs and promoted their apoptosis.

### Effect of *NCAPG* on proliferation and apoptosis of BMECs

**Figure 7:**
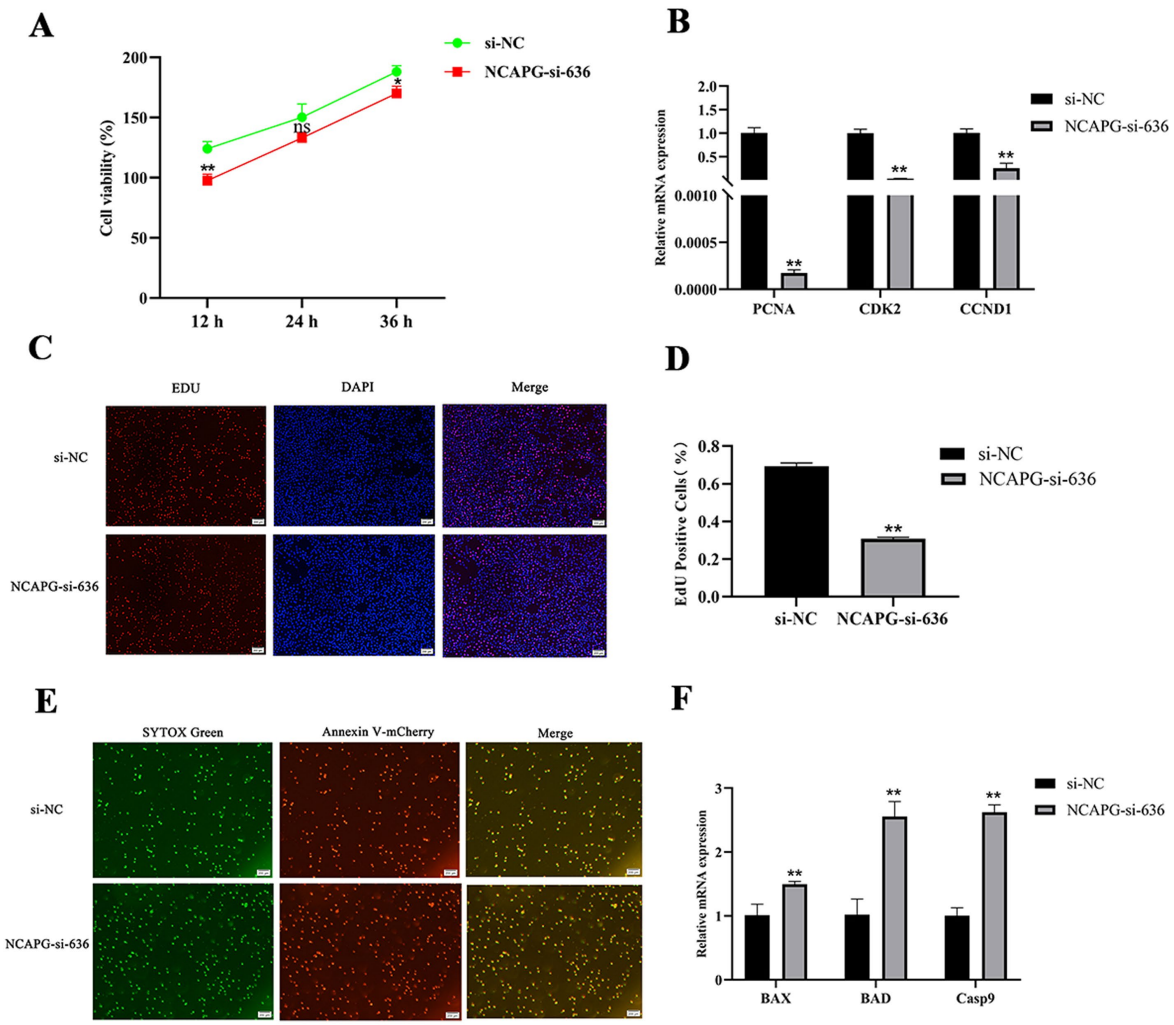
Effect of *NCAPG* on proliferation and apoptosis of BMECs. **(A)** Cell viability was detected by CCK8 after interfering with *NCAPG*; **(B)** the expression level of proliferation marker genes was detected after interfering with *NCAPG*; **(C,D)** cell proliferation was detected by EDU after interfering with *NCAPG* for 48 h and the percentage of EDU-labeled positive BMECs was counted by Image J software; **(E)** Cell apoptosis was detected after interfering with *NCAPG* for 48 h using Annexin V-mCherry/SYTOX Green to detect apoptosis; **(F)** Expression levels of apoptosis marker genes were detected after interference with *NCAPG*. Differences between two groups were compared using a two-tailed Student’s *t*-test, and comparisons between multiple groups were analyzed by one-way ANOVA. ** indicates highly significant differences (*p* < 0.01) and * indicates significant differences (*p* < 0.05).

To explore the effects of *NCAPG* on the proliferation and apoptosis of BMECs. CCK8 results showed (Figure 7A), *NCAPG*-si-636 highly significantly inhibited cell viability at 12 h (*p* < 0.01) and significantly inhibited cell viability at 36 h (*p* < 0.05). qRT-PCR results showed that compared with si-NC, *NCAPG*-si-636 highly significantly inhibited the expression of *PCNA*, *CDK2* and *CCND1* (*p* < 0.01) (Figure 7B); moreover, EDU results showed that *NCAPG*-si-636 could highly significantly inhibit the percentage of positive cell proliferation compared with si-NC (*p* < 0.01) (Figures 7C,D). The results of the apoptosis assay showed that the number of apoptotic BMECs was significantly increased after the knockdown of *NCAPG* (Figure 7E), and *NCAPG*-si-636 could extremely significantly up-regulate the expression levels of *BAX*, *BAD*, and *Casp9* compared with si-NC (*p* < 0.01) (Figure 7F). In summary, interference with *NCAPG* inhibited the proliferation and viability of BMECs and promoted their apoptosis.

### *SPP1* and *NCAPG* regulate casein synthesis in BMECs

**Figure 8:**
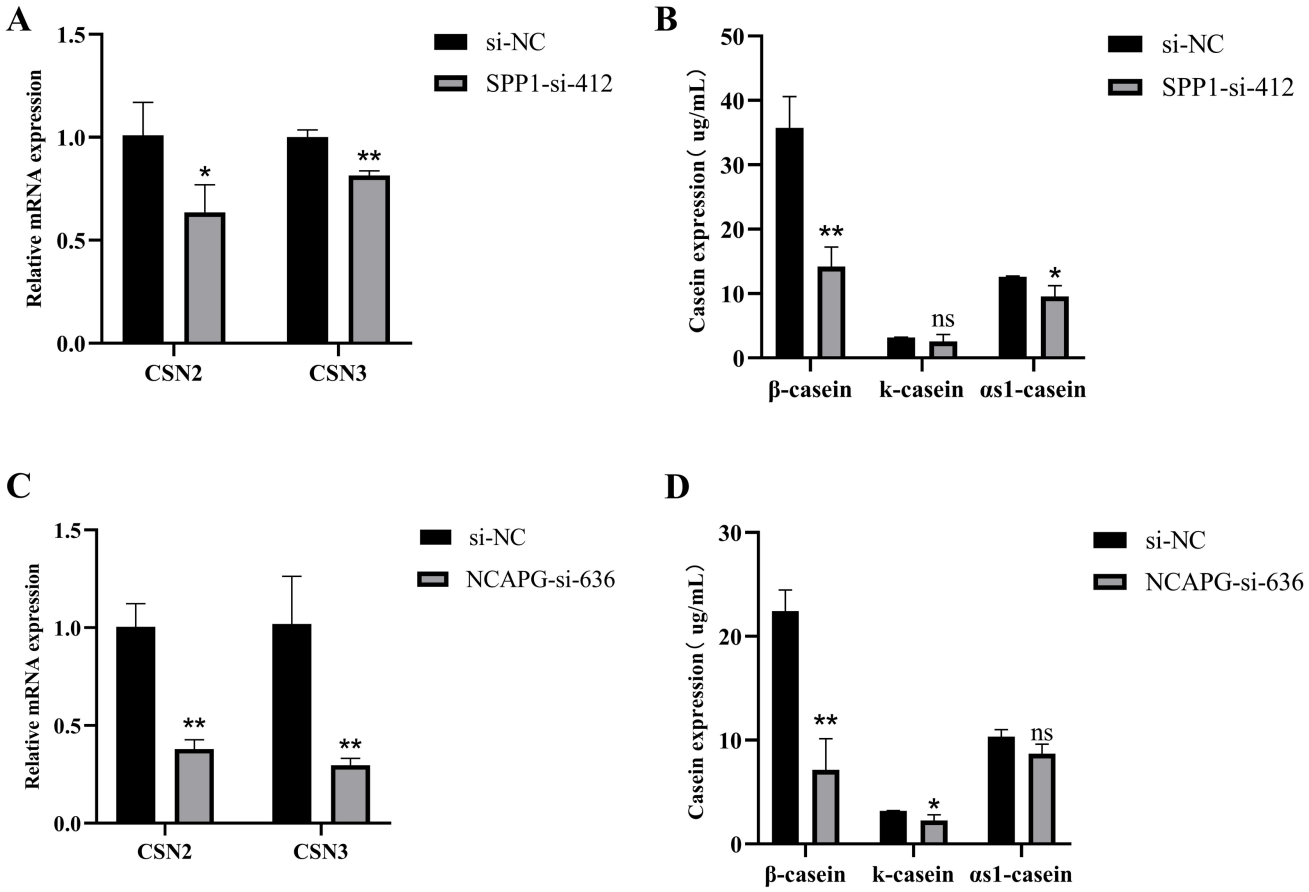
*SPP1* and *NCAPG* regulate casein synthesis in BMECs. **(A)** qRT-PCR to detect the expression of CSN2 and CSN3 after interfering with *SPP1*; **(B)** Detection of expression levels of *β*-casein, *κ*- casein and αs1- casein after interference with *SPP1*; **(C)** qRT-PCR to detect the expression of CSN2 and CSN3 after interfering with *NCAPG*; **(D)** Detection of expression levels of β-casein, κ- casein and αs1- casein after interference with *NCAPG*. Differences between two groups were compared using a two-tailed Student’s *t*-test, and comparisons between multiple groups were analyzed by one-way ANOVA. ** Indicates highly significant differences (*p* < 0.01) and * indicates significant differences (*p* < 0.05).

To further investigate the effects of *SPP1* and *NCAPG* on the secretion of casein by BMECs, after transfected, qRT-PCR results showed that interference with *SPP1*, compared with si-NC, significantly reduced the *CSN2* expression level (*p* < 0.05) and highly significantly reduced the expression level of *CSN3* (*p* < 0.01) (Figure 8A). Compared with si-NC, the expression level of *β*-casein was highly significantly reduced (*p* < 0.01), the expression level of *κ*-casein tended to be reduced, but the difference was not significant (*p* > 0.05), and the expression level of *α*-casein was significantly reduced (*p* < 0.05) (Figure 8B). Interference with *NCAPG* extremely significantly reduced the expression levels of CSN2 and CSN3 compared with si-NC (*p* < 0.01) (Figure 8C). Compared with si-NC, the expression level of β-casein was extremely significantly reduced (*p* < 0.01), the expression level of κ-casein was significantly reduced (*p* < 0.05), and the expression level of αs1-casein tended to be reduced, but the difference was not significant (*p* > 0.05) (Figure 8D). In summary, interference with *SPP1* and *NCAPG* decreased casein secretion in BMECs.

### *SPP1* and *NCAPG* regulate triglyceride synthesis in BMECs

**Figure 9:**
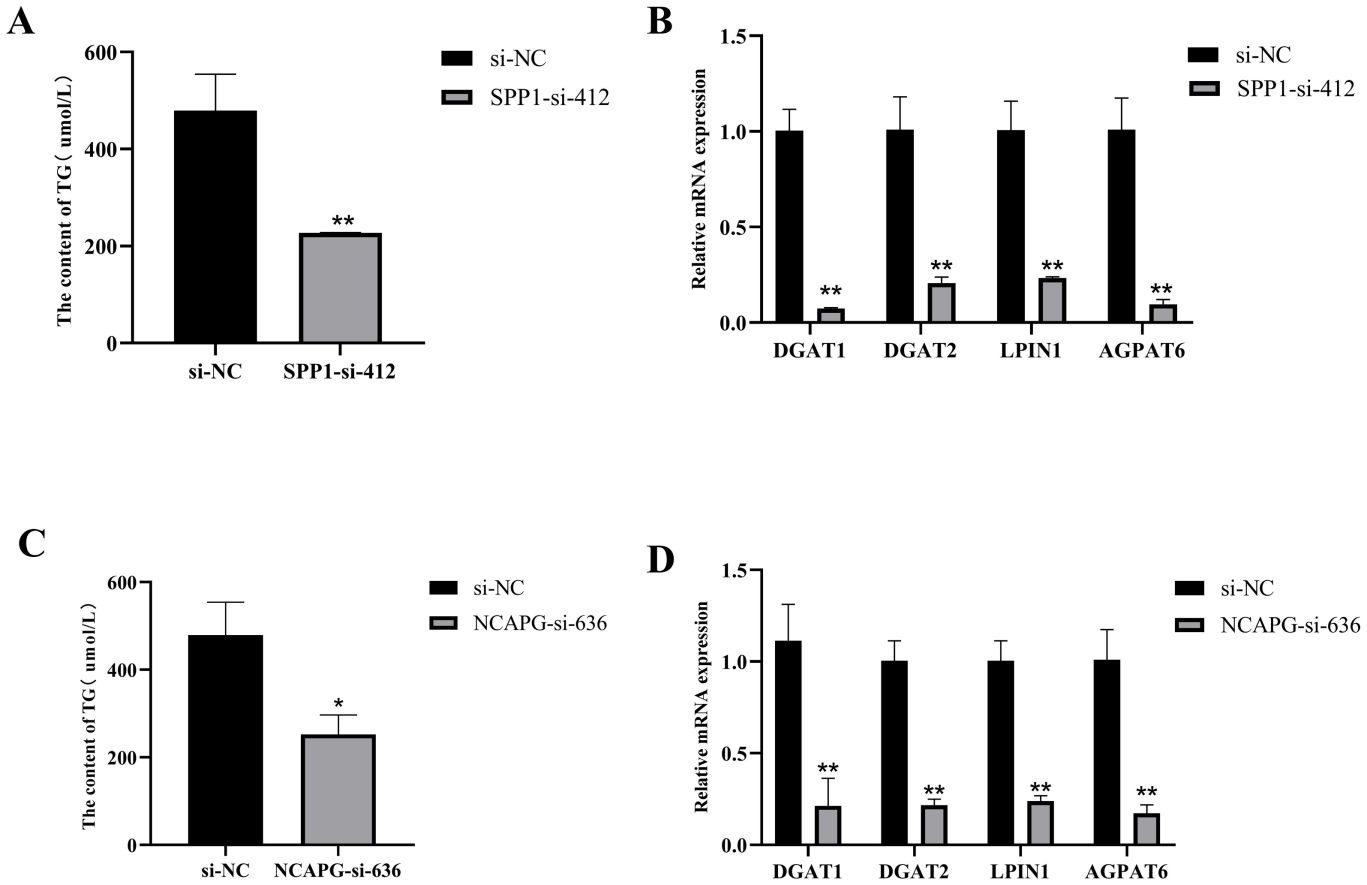
*SPP1* and *NCAPG* regulate triglyceride synthesis in BMECs. **(A)** Interference with *SPP1* to detect TG content; **(B)** qRT-PCR to detect the expression of DGAT1, DGAT2, LPIN1, and AGPAT6 after interference with *SPP1*; **(C)** Interference with *NCAPG* to detect TG content; **(D)** qRT-PCR to detect the expression of DGAT1, DGAT2, LPIN1, and AGPAT6 after interference with *NCAPG* expression of DGAT1, DGAT2, LPIN1 and AGPAT6. Differences between two groups were compared using a two-tailed Student’s *t*-test, and comparisons between multiple groups were analyzed by one-way ANOVA. ** Indicates highly significant differences (*p* < 0.01) and * indicates significant differences (*p* < 0.05).

To further investigate the effects of *SPP1* and *NCAPG* on triglyceride synthesis in BMECs, after transfected, which showed that interference with *SPP1* highly significantly reduced the expression level of TG compared to si-NC (*p* < 0.01), and highly significantly reduced the TG synthesis marker genes *DGAT1*, *DGAT2*, *LPIN1* and *AGPAT6* expression levels (*p* < 0.01) (Figures 9A,B). Interference with *NCAPG* significantly reduced the expression level of TG (*p* < 0.05) and highly significantly reduced the expression level of the TG synthesis marker genes *DGAT1*, *DGAT2*, *LPIN1*, and *AGPAT6* compared with si-NC (*p* < 0.01) (Figures 9C,D). Taken together, interfering with *SPP1* and *NCAPG* inhibited milk fat synthesis in BMECs.

## Discussion

Identification of SNPs in genes associated with milk production traits is essential to improve the prediction of the genetic breeding value of animals for genetic improvement of livestock production traits (19). In our previous study, *SPP1* and *NCAPG* were screened as candidate genes affecting milk production traits (5). In this study, a total of two SNPs in the *SPP1* gene and four SNPs in the *NCAPG* gene were identified. All SNPs were found to be significantly or highly significantly associated with at least one milk production trait. Meanwhile, the results of haplotype association analysis were generally consistent with the results of single marker association analysis, indicating that *SPP1* and *NCAPG* had a large genetic effect on milk production traits, and the SNPs identified and characterized in both *SPP1* and *NCAPG* genes were located in the exon region. Existing studies have shown (20) that exons are capable of influencing transcript splicing and thus protein sequences and individual phenotypes. With the continuous progress of technology, molecular marker-assisted breeding has become one of the technologies that have attracted much attention and application in the field of livestock and poultry production. This technology utilizes molecular marker technology and the principles of genetics to quickly and accurately screen out livestock breeds with excellent genetic characteristics, avoiding the cumbersome breeding process and long breeding cycle in traditional breeding methods.

*SPP1* is expressed in many tissues and secreted into body fluids, including blood, milk and urine and it plays an important role in tissue homeostasis, remodeling, immune regulation and stress response (21). The results of tissue expression profiling in this study showed that *SPP1* and *NCAPG* were expressed at the highest level in mammary tissues, which is hypothesized to play an important role in the regulation of lactation in dairy cows. It was shown (22) that among the genes in the microsatellite BM143 region of bovine chromosome 6, *SPP1* had the highest linkage disequilibrium effect on milk protein percentage, and that *SPP1* is a key candidate gene affecting milk protein percentage. Schnabel et al. (14) examined a population of 3,147 Holstein bulls from 45 half-sibling families for polymorphism in the *SPP1* gene and found four polymorphic loci, of which the OPN3907 mutation locus was the causal mutation affecting milk protein percentage. Leonard et al. (23) investigated *SPP1* polymorphisms and found that the fourth intron C/T mutation was significantly associated with milk fat percentage and milk protein percentage, with the C allele significantly increasing milk fat percentage and milk protein percentage. Matsumoto et al. (24) identified SNPs in the *SPP1* gene that were associated with beef quality, with SNPsg.58675C > T potentially affecting carcass weight by affecting the function, structure, or post-translational modification of *SPP1* to influence carcass weight. In this study, *SPP1*-g. 36,700,265 C > T was found to affect milk protein percentage highly significantly (*p* < 0.01), with TT genotypes of cows having a significantly higher milk protein percentage than CC and CT genotypes. *SPP1*-g. 36,693,596 C > A was found to affect milk protein percentage significantly (*p* < 0.05), with CC genotypes of cows having a significantly higher milk protein percentage than AA and CA genotypes (*p* < 0.05). A haplotype is a set of alleles inherited on a single chromosome containing DNA sequences of multiple SNPs. Haplotypes can distinguish the genetic information of different parental chromosomes, reveal the combination and inheritance of different genetic loci on a single chromosome or a specific single chromosome region, and help to explore the heterozygous dominance of animals. Under the dominant haplotype, *SPP1*-g. 36,693,596 C > A was a purebred CC and *SPP1*-g. 36,700,265 C > T was a purebred TT, which was consistent with single-marker association analysis. Our results indicated that this haplotype block significantly affected milk protein percentage. The haplotype block consisting of the four SNPs of *NCAPG* significantly affected milk protein percentage and highly significantly affected 305-days milk protein yield and 305 days milk yield. It was further inferred that *SPP1* and *NCAPG* genes may play important roles in milk fat metabolism and protein synthesis in dairy cows. The genetic markers obtained from our screening can effectively identify economically important traits in dairy cattle, which will lay the foundation for the process of genetic improvement of dairy cattle.

To further reveal the effects of *SPP1* and *NCAPG* genes on the proliferation and apoptosis of BMECs. We interfered with *SPP1* and *NCAPG* to detect the effects on cell viability, proliferation and apoptosis. *SPP1*, as a multifunctional protein, is involved in the regulation of many diseases by promoting inflammatory responses, cell proliferation and migration, and is closely related to the development of fatty liver and hepatocellular carcinoma (25). Studies have shown that knockdown of *SPP1* in combination with radiation therapy promotes apoptosis, inhibits the expression of downstream genes, and reduces cell viability in breast cancer cells (26). *SPP1* activates the PI3K/Akt pathway, promotes cell proliferation, and inhibits apoptosis (27). Consistent with the present study, interference with *SPP1* inhibited the proliferation and viability of BMECs, promoted their apoptosis, and suppressed the expression of *PCNA*, *CDK2*, and *CCND1*, and the trend of apoptosis marker genes was reversed. It was further verified that *SPP1* affects the proliferation and apoptosis of BMECs.

Studies have shown (28) that interference with *NCAPG* can inhibit the growth, proliferation and invasion of ovarian cancer cells (OC) by promoting the activation of the p38 MAPK signaling pathway, and it blocks the cell cycle and promotes apoptosis in the G2 and S phases. *NCAPG*, an oncogene of HCC (hepatocellular carcinoma), promotes cell proliferation and inhibits cell apoptosis (29). Studies have reported an association between apoptosis and *NCAPG* or cohesin I deletion. In zebrafish, *NCAPG* mutations resulted in increased genomic imbalance and increased retinal apoptosis (30). Knockdown of *NCAPG* during myogenic differentiation of fetal bovine adult myoblasts promotes apoptosis (16). However, to date, no study has demonstrated a regulatory effect of *NCAPG* on BMECs. Our findings are consistent with those of previous studies that interference with *NCAPG* extremely significantly upregulates the expression levels of *BAX*, *BAD*, and *Casp9*, inhibits the proliferation and viability of BMECs, and promotes their apoptosis. Most mammary epithelial cells are secretory cells that undergo functional differentiation during pregnancy, and the number of BMECs is inextricably linked to milk production and quality (31). It is hypothesized that *SPP1* and *NCAPG* promote the proliferation of BMECs inhibiting their apoptosis thereby affecting the milk fat and milk protein content of milk.

Triglycerides (TG) are the main components of milk fat, accounting for 98% of the total lipid content. *DGAT1* and *DGAT2* are mainly used to regulate TAG synthesis. and store excess fatty acids in lipid droplets (LD) (32). *SPP1* is an inflammatory cytokine highly upregulated in obese adipose tissue and it has been repeatedly shown to functionally promote obesity and regulate lipid synthesis (33). In a porcine model of NASH (nonalcoholic steatohepatitis), *SPP1* gene expression was significantly and positively correlated with lipid droplet area and inflammatory response and was significantly reduced when NASH was reversed (34). *NCAPG* I442M is associated with postnatal weight gain and lipid deposition in cattle as derived from data on the bovine *NCAPG* gene and comparative information from the human and mouse genomes (35–37), and arginine and its metabolite symmetric dimethylarginine were significantly associated with the *NCAPG* I442M mutation (38). In this study, interference with both *SPP1* and *NCAPG* inhibited TAG synthesis in BMECs, and the expression levels of the marker genes *DGAT1*, *DGAT2*, *LPIN1*, and *AGPAT6* were all highly significantly reduced. Consistent with the trend in the above findings, the results lay the foundation for subsequent studies on the mechanisms by which *SPP1* and *NCAPG* regulate milk fat metabolism.

Casein is the main protein in milk, accounting for about 80% of milk protein. It mainly consists of four types: *β*-casein, *κ*-casein, αs1-casein and αs2-casein, and has different structures and functions. Milk protein synthesis is a complex biological process, and studies have shown that miR-139 may inhibit β-casein synthesis through GHR and IGF1R signaling in BMECs (39). Wang et al. (40) found that Pten down-regulated *β*-casein secretion by mammary epithelial cells in dairy cows. In this study, we found that the expression levels of β-casein, *κ*-casein and αs1-casein were reduced after interference with *SPP1* and *NCAPG*. Among them, the level of β-casein was reduced most significantly. Polymorphisms of both *SPP1* and *NCAPG* were significantly correlated with milk protein percentage. Thus *SPP1* and *NCAPG* may affect milk protein synthesis and lactation in BMECs.

In future studies, there is a need to validate the findings in different herds and conduct mechanistic studies. As well as the inclusion of more diverse samples of Holstein cows from different regions to validate the reliability and generalizability of the findings. Further studies on the fine molecular regulatory mechanisms by which *SPP1* and *NCAPG* affect milk production traits are also needed. The use of technologies such as CRISPR-Cas9 could provide deeper insights.

## Conclusion

In this study, two SNPs and one haplotype block of the *SPP1* gene and four SNPs and one haplotype block of the *NCAPG* gene were screened and characterized. These SNPs were significantly associated with milk protein percentage. Constitutive haplotype blocks were correlated with milk production traits such as milk protein percentage. In addition, interference with *SPP1* and *NCAPG* affected cell viability, proliferation, and apoptosis by regulating the expression of marker genes (*PCNA*, *CDK2*, *CCND1*, *BAX*, and *BAD*, etc.), and inhibited the expression levels of milk lipid marker genes (*DGAT1*, *DGAT2*, *LPIN1*, and *AGPAT6*) reduced triglycerides, β-casein, κ-casein, κ-casein, and κ-casein in BMECs and αs1-casein synthesis. Taken together, *SPP1* and *NCAPG* may be key candidate genes influencing marker-assisted selection in dairy cattle breeding.

## Data Availability

The original contributions presented in the study are included in the article/Supplementary material, further inquiries can be directed to the corresponding author/s.

## Footnotes

^1^ https://www.ncbi.nlm.nih.gov/
